# Pregabalin: A range of misuse‐related unanswered questions

**DOI:** 10.1111/cns.13115

**Published:** 2019-03-04

**Authors:** Fabrizio Schifano, Stefania Chiappini

**Affiliations:** ^1^ Psychopharmacology, Drug Misuse and Novel Psychoactive Substances Research Unit, School of Life and Medical Sciences University of Hertfordshire Hatfield UK

Although initially marketed as having a low abuse potential, further recent, large‐scale, papers^1^ have once again convincingly emphasized the pregabalin potential of misuse. However, after a decade of intensive pregabalin‐focused studies, still a few issues remain open.

Indeed, the first to explore is why a potential for misuse for pregabalin is there. Is it related to a direct/indirect dopaminergic activity similar to remaining drugs of misuse? Why is pregabalin being typically misused in combination with opiates/opioids? Pregabalin is a known inhibitor of α2δ‐subunit‐containing voltage‐dependent calcium channels.^2^ The entry of calcium ions into neurons allows the process of vesicle fusion with the cell membrane, which facilitates proper release of neurotransmitters. Ultimately, the potent binding of pregabalin at the calcium channel will reduce the release of excitatory molecules (eg, glutamate, noradrenaline, and substance P, but not dopamine), acting against aberrant neuronal stimulation.^2^ Several addictive drugs have in common that they increase the extracellular dopaminergic activity in the mesolimbic reward system.^3^ In rats, conditioned place preference was, however, induced only with high intraperitoneal (but not oral) pregabalin doses, restricting the ability to develop a substantial addictive power.^4^ Accordingly, patients reported pleasant stimulation and euphoria when using supratherapeutic/mega (eg 1500‐12 000 mg) pregabalin dosages.^5^, ^6^ Hence, one could wonder if there may be a different/unclear range of neurotransmitter involvement, and receptors’ activation intensity, in high/very high pregabalin dosage ingestion. Overall, similar to what was observed with a range of further medications (eg, venlafaxine, bupropion, quetiapine, and loperamide),^6^ it has been suggested that gabapentinoids may induce a “liking” (euphoric high) subjective feeling, due to their gamma‐aminobutyric acid (GABA)‐mimetic action, but more limited levels of “wanting”/behavioral dependence.^7^ Cairns et al^1^ have identified that pregabalin was typically ingested in combination with opiates/opioids. Indeed, opioids may well be prescribed to potentiate gabapentinoid analgesic effects for treating specific medical conditions/intractable pain.^5^ However, pregabalin may clinically counteract the opioids’ withdrawal effects while presenting as well with potentiating effects when given to mice with existing opioid levels.^8^

Second, it has been^1^ suggested that the misuse of pregabalin may typically be associated with a history of polydrug misuse. However, what is the potential for misuse among those with different drug use experiences and who may not increase dosages overtime? According to Bonnet and Scherbaum^7^ with respect to remaining recreational drugs of misuse, there is less evidence for gabapentinoids being misused in a long‐term manner. However, there are no formal data in the literature about clients who have voluntarily sought treatment for their pregabalin addiction or pregabalin relapses after detoxification.^7^ Although observations of behavioral dependence were described in patients who had no prior substance abuse history, these cases appeared to be quite rare.^7^ Kapil et al^9^ used an online survey to assess the self‐reported lifetime prevalence of misuse of GABA‐analogues in the younger and mid‐life UK general population and found that gabapentin and pregabalin were, respectively, being misused by 1.1% and 0.5% of the survey's respondents. Further valid prevalence data of gabapentinoid abuse from large‐scale structured interviews have recently been made. Snellgrove et al^10^ carried out a cross‐sectional study with some 253 addicts on a detoxification ward in southern Germany. They found that some 56% had used pregabalin at least once and that DSM‐IV dependence criteria were met by 11% of pregabalin users. Further systematic prevalence data from a cohort of 400 randomly selected elderly hospitalized population were provided by Cossmann et al^11^ A fifth of the cohort was found to be mildly dependent on nonopioid analgesics, but one case with a previous dependence on gabapentinoids was identified

Third, in many studies^1^, ^5^ it was unclear if pregabalin was most typically prescribed to those affected by anxiety conditions to either “boost” and/or to replace existing benzodiazepine prescriptions. Is the pregabalin state of mind different from that associated with benzodiazepines’ intake? Even though pregabalin is structurally related to GABA, and although there are not any known direct actions on GABA or its receptors, therapeutic doses of pregabalin are dose‐dependently associated with increase in extracellular GABA levels.^7^ Most likely, this drives the relaxation and euphoria which are reported at the commencement of prescribed pregabalin use.^7^ However, different from clonazepam, high‐dosage pregabalin has been anecdotally described as an “ideal psychotropic drug” for recreational purposes to achieve specific mindsets, including relaxation and disinhibition, for example, alcohol/GHB/benzodiazepine‐like effects mixed with euphoria; to achieve entactogenic feelings/dissociation; and to cope with opiate/opioid withdrawal.^10^, ^12^

Consistent with these concerns, the rate of pregabalin‐related ambulance attendances has recently increased^13^ and growing numbers of deaths have been associated with pregabalin misuse. These misusing levels mostly occur together with other sedatives, such as benzodiazepines, alcohol, and opioids.^13^, ^14^ In all these polydrug intoxication cases, pregabalin, while contributing in terms of overall central nervous system depression,^1^, ^6^ may well have worsened the observed clinical outcomes. However, one could still argue that at least in some cases, pregabalin may be identified as just an easily accessible molecule with a “liking” feature, and this issue may need more debate and research….’.

Both pregabalin and gabapentin have been reclassified as class C controlled substances in the UK. Nonetheless, in the US, pregabalin is designated as a Schedule V controlled substance, while gabapentin is a controlled substance only in some states (Tennessee, Kentucky). Moreover, in Australia, pregabalin and gabapentin are still classified as Prescription Only (Schedule 4) medications, meaning that, similar todrugs like statins and antibiotics, they are not associated with any special controls on supply or possession.^13^ It is here suggested that, whenever psychotropics are to be made available on prescription, a range of abuse liability‐focused and pre‐marketing laboratory testing may need to be carried out. These phase 3 studies should also assess how the new drug may interact with alcohol and/or other drugs. Also, post‐marketing surveillance studies should be encouraged to more accurately assess the true misusing potential of any psychotropic molecule. Physicians should be vigilant when prescribing drugs with a misuse/diversion potential and carefully evaluate the possibility that some clients (including people with a personal history of drug misuse or abuse) may be more vulnerable.

## DISCLOSURE STATEMENT

FS is a UK Advisory Council on the Misuse of Drugs (ACMD) member; and European Medicines Agency (EMA) Advisory Board (Psychiatry) member.
